# The impact of the Japanese clinical guidelines on the clinical management of patients with acute cholecystitis

**DOI:** 10.1007/s00534-013-0603-5

**Published:** 2013-04-09

**Authors:** Satoshi Shinya, Yuichi Yamashita, Tadahiro Takada

**Affiliations:** 1 Department of Gastroenterological Surgery, Fukuoka University School of Medicine, 7-45-1 Nanakuma, Jonan-ku, Fukuoka, 814-0180 Japan; 2 Department of Surgery, Teikyo University School of Medicine, 2-11-1 Kaga, Itabashi-ku, Tokyo, 173-8605 Japan

**Keywords:** Acute cholecystitis, Guidelines, Laparoscopic cholecystectomy, Questionnaire

## Abstract

**Background/purpose:**

The Japanese clinical guidelines for treating acute cholecystitis (AC), proposed in 2005, provide criteria not only for diagnosis, but also for management depending on the severity of the disease. The aim of this study was to assess how the Japanese guidelines for AC have impacted the clinical situation in Japan.

**Methods:**

A postal questionnaire was sent to the councillors of the Japanese Society of Abdominal Emergency Medicine three times to ascertain the impact of the Japanese guidelines for AC. We surveyed 291 councillors one year before publication of the guidelines (2004), 279 councillors one year after publication (2006), and 191 councillors six years after publication (2011).

**Results:**

The response rate was 72.5 % one year before publication of the guidelines, 51.9 % one year later and 69.1 % six years after publication.

Early cholecystectomy was used by 41.7 % of the respondents one year before publication, while 57.3 % of the respondents used this treatment one year after publication and 62.3 % of the respondents used it six years after publication. Laparoscopic cholecystectomy was used by 79.1 % of the respondents one year before the guidelines were published, while 87.3 % of the respondents used it one year after publication and 90 % of the respondents reported its use six years after publication.

**Conclusions:**

The Japanese guidelines for AC are increasingly used and have changed the clinical management of patients with AC. The use of early and laparoscopic cholecystectomy for treating patients with AC has been increasingly adopted in Japan.

## Introduction

Acute cholecystitis (AC) is a common complication of cholelithiasis and a frequent cause of abdominal pain requiring hospitalization. The incidence of mortality from AC is declining due to the introduction of advanced diagnostic tools for early detection, improvements in conservative treatments, including antibiotics, intravenous fluids, narcotics for pain management and surgical procedures [1–4]. Acute cholecystitis consists of various morbid conditions ranging from mild cases that are relieved by the oral administration of antibiotics or that resolve without the administration of antibiotics to severe cases complicated by biliary peritonitis, which requires a different treatment strategy [5]. The Japanese clinical guidelines for acute cholangitis and cholecystitis published in 2005 prior to the Tokyo guidelines for the management of acute cholangitis and cholecystitis (TG07) provide recommendations not only for diagnostic criteria, but also for management depending on the severity of AC [6]. To the best of our knowledge, no report has yet addressed the effects of these guidelines on the management of patients with AC. The aim of this study was to evaluate the chronological impact of the Japanese clinical guidelines for AC on the management of patients with AC in Japan.

## Methods

The names and addresses of the councillors of the Japanese Society of Abdominal Emergency Medicine were obtained from the office of the association three times. Information was collected for 447 councillors one year before guidelines publication (2004), for 437 councillors one year after guidelines publication (2006) and for 400 councillors six years after publication (2011). The same questionnaire [7] was sent to 291 councillors who were surgeons (excluding physicians, pediatric surgeons and other councillors at the same hospital) one year before publication (2004), 279 councillors one year after publication (2006) and 191 councillors six years after publication (2011). Six years after the Japanese guidelines were published, we questioned the councillors regarding the TG07 proposed in 2007. All data were collected at the Department of Gastroenterological Surgery of Fukuoka University Hospital. The questions asked in the questionnaire are shown in the appendix. The survey collected information on the hospital department initially receiving patients with AC, the timing of surgical management of AC and the surgical procedure used, including intraoperative cholangiography, in addition to a general questionnaire. In particular, the surgeons were asked whether they routinely opted for early cholecystectomy on the patient's first admission. All respondents were asked about their chosen method of cholecystectomy in the early and delayed stages of inflammation. The replies to the questionnaires were signed.

## Results

The response rate was 72.5 % one year before the publication of the guidelines, 51.9 % one year after publication and 69.1 % six years after publication.

### Total number of cholecystectomies performed in patients with AC per year (Questions 1, 2)

**Fig. 1:**
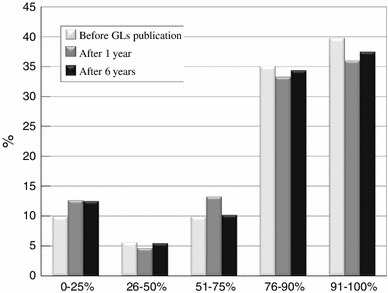
The population of patients who underwent laparoscopic cholecystectomy (Q1/Q2)

The total number of cholecystectomies performed in patients with AC per year for each time period are shown in Table 1. The rates of laparoscopic cholecystectomy for each time period of the postal questionnaire survey are shown in Fig. 1.

**Table 1:** Total number of cholecystectomies performed in patients with AC per year (cholecystectomies)

	0–12	13–25	26–50	51–100	100~
One year before GLs publication	111	60	28	11	1
Total 211	(52.6 %)	(28.4 %)	(13.3 %)	(5.2 %)	(0.5 %)
One year after GLs publication	76	43	22	4	0
Total 145	(52.4 %)	(29.7 %)	(15.1 %)	(2.8 %)	(0 %)
Six years after GLs publication	65	42	14	10	1
Total 132	(49.2 %)	(31.8 %)	(10.6 %)	(6.1 %)	(0.8 %)

### Management of acute cholecystitis (AC) (Questions 3–6)

**Fig. 2:**
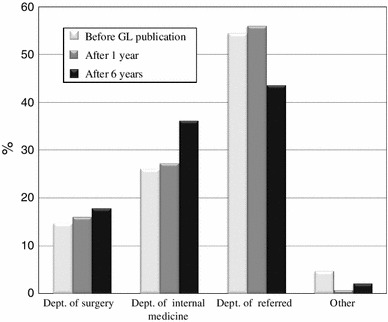
The hospital departments initially receiving patients with AC (Q3)

**Fig. 3:**
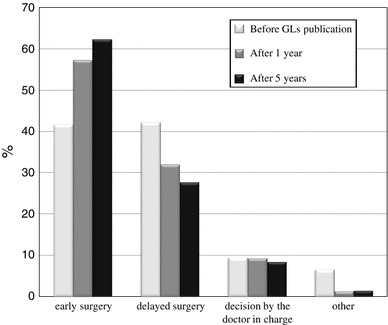
The percentage of patients with AC undergoing early or delayed cholecystectomy within 72 h of onset (Q4)

**Fig. 4:**
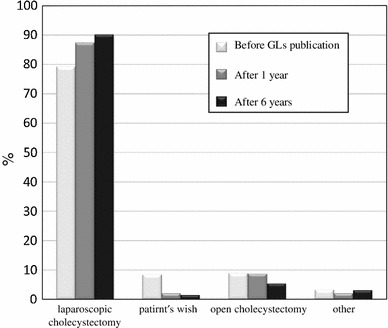
The cholecystectomy procedures recommended for patients with AC by Japanese surgeons (Q5)

**Fig. 5:**
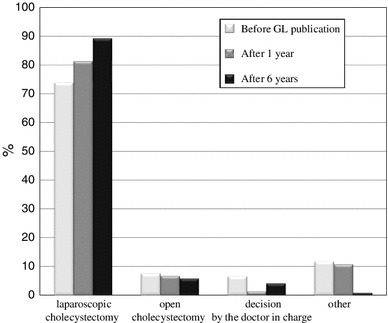
The cholecystectomy procedures recommended for patients with AC who have previously undergone percutaneous transhepatic gallbladder drainage (PTGBD) (Q6)

The types of hospital departments initially receiving patients with AC one year before guidelines publication (2004), one year after guidelines publication (2006) and six years after guidelines publication (2011) are shown in Fig. 2. As shown in Fig. 3, the use of early cholecystectomy was adopted by 41.7 % of the surgeons one year before publication, 57.3 % of the surgeons one year after publication and 62.3 % of the surgeons six years after publication. The use of delayed elective cholecystectomy was adopted by 42.2 % of the surgeons one year before publication, 32 % of the surgeons one year after publication and 27.7 % of the surgeons six years after publication. As shown in Fig. 4, when obtaining informed consent for surgical treatment during the first admission, the use of laparoscopic cholecystectomy was recommended by 79.1 % of the respondents one year before guidelines publication, 87.3 % of the respondents one year after publication and 90 % of the respondents six years after publication. The use of open cholecystectomy was recommended by less than 10 % of the respondents for each time period. As shown in Fig. 5, the use of laparoscopic cholecystectomy in patients with AC who had percutaneous transhepatic gallbladder drainage (PTGBD) was adopted by 73.9 % of the respondents one year before guidelines publication, 81.3 % of the respondents one year after publication and 89.3 % of the respondents six years after publication. The use of open cholecystectomy was adopted by 5 to 8 % of the respondents for each time period.

### Intraoperative cholangiography (Questions 7, 8)

**Fig. 6:**
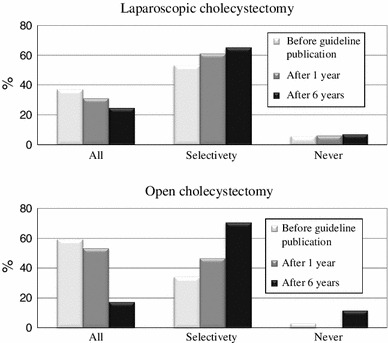
The adoption rates of intraoperative cholangiography in patients who undergo laparoscopic or open cholecystectomy (Q7/Q8)

The rate of performing intraoperative cholangiography in patients who undergo laparoscopic and open cholecystectomy are shown in Fig. 6.

### Diagnostic imaging techniques for AC considered to be indispensable preoperatively (Question 9)

**Fig. 7:**
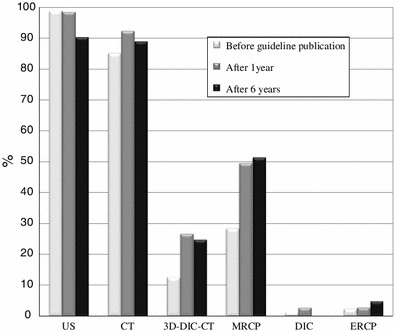
The preoperative imaging modalities reported to be indispensable for acute cholecystitis diagnosis (Q9)

As shown in Fig. 7, ultrasound (US) was used by over 90 % of the respondents during all time periods. Computed tomography (CT) was used by about 90 % of the respondents during all time periods. Magnetic resonance cholangiopancreatography (MRCP) was used by less than 30 % of the respondents one year before publication of the guidelines, and by about 50 % of the respondents at one year and six years after the guidelines were published. Three-dimensional drip infusion cholangiography-CT (3D-DIC-CT) was used by 12 % of the respondents one year before publication, and by about 25 % of the respondents at one year and six years after publication. Drip infusion cholangiography (DIC) was used by a very small percentage of the respondents at one year before and one year after publication, and by 0 % of the respondents at six years after publication. Endoscopic retrograde cholangiopancreatography (ERCP) was used by 2 to 5 % of all respondents for all time periods.

### Utilization of the guidelines for AC (Questions 10–13)

**Fig. 8:**
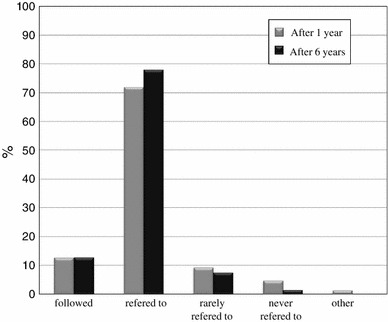
The percentage of surgeons who used the Japanese guidelines (Q10)

**Fig. 9:**
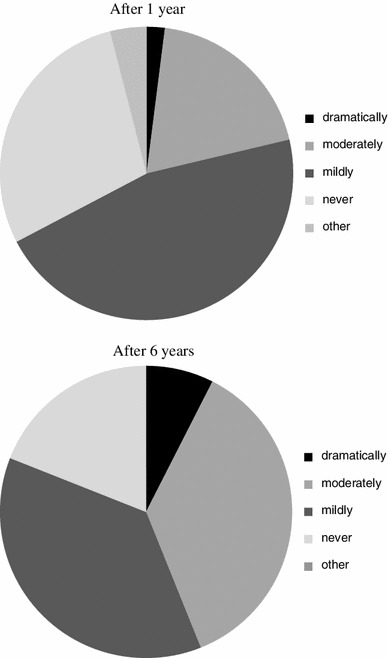
The impact of the guidelines on the clinical management of acute cholecystitis (Q11)

**Fig. 10:**
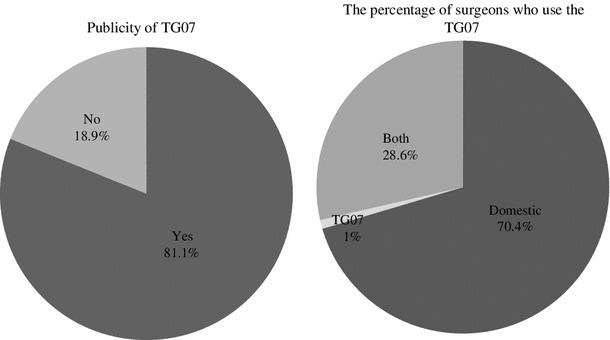
The percentage of surgeons who used the Tokyo guidelines (Q12/13)

As shown in Fig. 8, regarding the management of patients with AC, about 13 % of the surgeons strictly followed the guidelines, and over 70 % at least referred to the guidelines. On the other hand, less than 10 % only rarely referred to the guidelines and less than 5 % never referred to the guidelines at all, at both one and six years after they were published. As shown in Fig. 9, regarding the clinical management of patients with AC, 2 % of the surgeons surveyed felt it had “dramatically changed” and 19.3 % felt it had “moderately changed,” while 46 % felt it had “mildly changed” and 28.7 % felt it had “never changed” one year after guidelines publication. Six years after publication, 7.6 % of the surgeons felt that management had “dramatically changed” and 36.4 % felt that it had “moderately changed,” while 37.1 % felt that it had “mildly changed” and 19 % felt that it had “never changed.” As shown in Fig. 10, at six years after publication, over 80 % of the surgeons were aware of the TG07 that had been proposed as part of the international guidelines for AC in 2007. The Japanese guidelines were used by 70.4 % of the surgeons, the TG07 were used by 1 % of the surgeons and both guidelines were properly used by 28.6 % of the surgeons.

## Discussion

The introduction of advanced diagnostic modalities for early detection and prompt administration of treatment that is adequate for the severity of AC can reduce the incidence of mortality and morbidity associated with AC. For this purpose, the Japanese clinical guidelines for acute cholangitis and cholecystitis published in 2005 provide a framework for the diagnosis and management of AC in the clinical setting based on the best available evidence and the consensus of Japanese experts [6].

Our postal survey revealed changes in utilization of the guidelines for AC and the effects of the guidelines on clinical management. Of the respondents, 12.7 % of the Japanese surgeons followed the guideline and 72 % referred to the guidelines; therefore, a total of 84.7 % of the Japanese surgeons used the guidelines one year after publication. Six years after publication, 12.9 % of the surgeons followed the guidelines and 78 % referred to the guidelines for a total of 90.9 % of Japanese surgeons who reportedly used the guidelines. In addition, more Japanese surgeons felt that the guidelines brought about changes in the clinical management of AC six years after publication compared to only one year after publication. These results showed increased implementation of the guidelines over time.

Our postal survey showed that the use of early cholecystectomy has been gradually increasing. Before the guidelines were published, the adoption rates of early and delayed elective cholecystectomy were almost the same, while the adoption rate of early cholecystectomy was almost twice that of delayed elective cholecystectomy six years after guidelines publication. Japanese surgeons have recognized the usefulness of early cholecystectomy; however, the procedure has remained unpopular in Japan [7]. Several studies have shown a lack of available experienced surgeons due to the pressures of other clinical and management commitments, and the limited availability of surgical theater space has resulted in a low incidence of routine early cholecystectomy as a treatment for acute cholecystitis in the United Kingdom [8, 9]. Although our questionnaire did not include items examining the reasons why early cholecystectomy remains unpopular, the reasons may be similar to those in the United Kingdom. In addition, logistical difficulties such as a lack of available anesthetists and an imbalance in the distribution of anesthetists among Japanese hospitals currently exist. These factors might have restricted the ability of Japanese surgeons to perform early cholecystectomy in patients with AC.

Regarding surgical technique, this survey showed that the use of laparoscopic cholecystectomy has been gradually adopted by Japanese surgeons. Previously, AC was considered to be a relative contraindication of urgent LC because several studies of urgent LC for AC often showed technical difficulties, greater morbidity rates, prolonged operation times and higher conversion rates to open surgery compared with elective LC [10, 11].

Our postal survey also showed that the use of open cholecystectomy in patients with AC who have preoperatively undergone percutaneous transhepatic gallbladder drainage (PTGBD) has been decreasing, while the use of laparoscopic cholecystectomy has been gradually increasing. Recently, the management of patients with AC has evolved with increases in laparoscopic experience, as urgent LC can be performed safely in patients with AC with low rates of morbidity and conversion to open surgery [12–15]. This may be the one of the reasons why the use of urgent LC has been increasing.

In emergency situations, proper and adequate imaging modalities should be administered in order to confirm the diagnosis and severity of the disease. This could result in the use of surgical treatment without missing the proper timing. This study identified the indispensable procedures used for the preoperative diagnosis of AC preferred by Japanese surgeons. In each of the three surveys, US and CT were reported to be indispensable preoperative diagnostic imaging techniques, while ERCP and DIC were not often used. In particular, no Japanese surgeons used DIC six years after guidelines publication. MRCP and 3D-DIC-CT have been increasingly used. These trends suggest that Japanese surgeons attempt to reduce the incidence of complications of bile duct injury by confirming the stricture and anomaly of the bile ducts when possible.

This survey showed that intraoperative cholangiography is selectively performed in patients who undergo laparoscopic or open cholecystectomy. It has been speculated that this trend is caused by the increased utilization of preoperative MRCP and 3D-DIC-CT for assessment of the bile ducts.

This survey also evaluated awareness of the TG07 proposed as international guidelines in 2007, which provide recommendations for diagnostic criteria and management depending on the severity of acute cholecystitis [16]. Of the Japanese surgeons included in this study, 81.1 % were aware of TG07; however, these guidelines were not primarily used. The TG07 were properly used in association with the Japanese guidelines by 28 % of the Japanese surgeons.

This survey indicated that many Japanese surgeons use the guidelines, as shown in the increased use of urgent LC. Currently, it is time to revise the Japanese guidelines. Conducting a survey of the changes in the management of patients with AC induced by the guidelines in their present state can help to best create new guidelines. Unless surgeons close the gap between the guidelines and practice in the clinical setting, they will not be able to communicate with each other effectively. Japanese surgeons use two sets of guidelines for treating AC. Conducting a repeat survey will be necessary in order to determine any changes in the timing and approach to the surgical management of patients with AC and the dissemination of the guidelines.

## Appendix

Questionnaire

## Abbreviations

AC: Acute cholecystitis
TG 07: Tokyo guidelines for the management of acute cholangitis and cholecystitis
